# Effect of Stress Signals and *Ib-rolB/C* Overexpression on Secondary Metabolite Biosynthesis in Cell Cultures of *Ipomoea batatas*

**DOI:** 10.3390/ijms232315100

**Published:** 2022-12-01

**Authors:** Elena A. Vasyutkina, Yulia A. Yugay, Valeria P. Grigorchuk, Olga V. Grishchenko, Maria R. Sorokina, Yulia L. Yaroshenko, Olesya D. Kudinova, Varvara D. Stepochkina, Victor P. Bulgakov, Yury N. Shkryl

**Affiliations:** 1 Federal Scientific Center of the East Asia Terrestrial Biodiversity, Far East Branch, Russian Academy of Sciences, Vladivostok 690022, Russia; 2 Advanced Engineering School, Institute of Biotechnology, Bioengineering and Food Systems, Far Eastern Federal University, 10 Ajax Bay, Russky Island, Vladivostok 690922, Russia

**Keywords:** sweet potato, *Agrobacterium rhizogenes*, *rol* genes, abiotic stress, polyphenols, naturally transgenic plants

## Abstract

*Ipomoea batatas* is a vital root crop and a source of caffeoylquinic acid derivatives (CQAs) with potential health-promoting benefits. As a naturally transgenic plant, *I. batatas* contains cellular T-DNA (cT-DNA) sequence homologs of the *Agrobacterium rhizogenes* open reading frame (ORF)14, ORF17n, rooting locus (Rol)B/RolC, ORF13, and ORF18/ORF17n of unknown function. This study aimed to evaluate the effect of abiotic stresses (temperature, ultraviolet, and light) and chemical elicitors (methyl jasmonate, salicylic acid, and sodium nitroprusside) on the biosynthesis of CQAs and cT-DNA gene expression in *I. batatas* cell culture as a model system. Among all the applied treatments, ultraviolet irradiation, methyl jasmonate, and salicylic acid caused the maximal accumulation of secondary compounds. We also discovered that *I. batatas* cT-DNA genes were not expressed in cell culture, and the studied conditions weakly affected their transcriptional levels. However, the *Ib-rolB/C* gene expressed under the strong 35S CaMV promoter increased the CQAs content by 1.5–1.9-fold. Overall, our results show that cT-DNA-encoded transgenes are not involved in stress- and chemical elicitor-induced CQAs accumulation in cell cultures of *I. batatas*. Nevertheless, overaccumulation of RolB/RolC transcripts potentiates the secondary metabolism of sweet potatoes through a currently unknown mechanism. Our study provides new insights into the molecular mechanisms linked with CQAs biosynthesis in cell culture of naturally transgenic food crops, i.e., sweet potato.

## 1. Introduction

*Ipomoea batatas* (sweet potato) is a valuable and nutritious root crop originating in Central and South America and extensively cultivated in tropical and warm temperate climates. *I. batatas* is among the seven most essential food crops in the world and the third most important root crop after potato and cassava, with more than 100 million tons of annual production produced on about 8.1 million hectares, mainly in the developing countries of East Asia and Africa [1,2,3]. It is a primary food in many developing countries and is reckoned as one of the main food crops in more than 50 countries [4]. Additionally, the accumulated data indicate that *I. batatas* is a potential source of natural compounds accountable for antioxidant, antidiabetic, wound healing, antiulcer, antibacterial, and antimutagenic activities [1,5,6,7].

The major secondary metabolites produced by sweet potato plants are caffeoylquinic acids (CQAs), namely 5-O-caffeoylquinic acid (5-CQA), 3-caffeoylquinic acid (3-CQA), 4-caffeoylquinic acid (4-CQA), 3,4-di-O-caffeoylquinic acid (3,4-diCQA), 3,5-di-O-caffeoylquinic acid (3,5-diCQA), 4,5-di-O-caffeoylquinic acid (4,5-diCQA), and 3,4,5-tri-O-caffeoylquinic acid (3,4,5-triCQA) [8,9]. CQAs are phenolic compounds formed during the esterification process from caffeic and quinic acids through the phenylpropanoid pathway. The primary genes involved in the biosynthetic process are contemplated to be phenylalanine ammonia-lyase (PAL), cinnamate 4-hydroxylase (C4H), 4-coumarate-CoA ligase (4CL), 4-hydroxycinnamoyl-CoA: shikimate/quinate hydroxycinnamoyl transferase (HCT/HQT), p-coumaroyl shikimic acid/quinic acid 3′-hydroxylase (C3´H), and isochlogogenate synthase (ICS) [10]. The specific CQAs composition relies on the exact cultivar [8].

Plants produce polyphenols in optimal living conditions, but they are primarily required for adaptation to survival under stress conditions [11]. The phenylpropanoid biosynthetic pathway is activated as a consequence of extreme temperatures, drought, salinization, UV-radiation, light intensity, toxic metals, or wounding, causing the accumulation of special metabolites possessing antioxidant properties, including chlorogenic acid and its derivatives. Therefore, salt stress stimulated CQAs synthesis in the *Lonicera japonica* leaves [12], drought intensified the accumulation of phenolic acids and flavonoids in *Amaranthus tricolor* [13], UV increased the concentration of phenolic compounds, including phenolic acids, in the leaves of lettuce [14], and several abiotic stresses caused secondary metabolism, particularly mono-CQAs in tomato leaves [15]. Sucrose-induced osmotic stress has been found to manage the phenolic acids, rosmarinic acid, chlorogenic acid, and caffeic acid production in the *Eryngium planum* callus [16]. It was also observed that diCQAs displayed stronger free radical scavenging activities than monoCQAs [17]. Furthermore, applying exogenous polyphenolic compounds raised the stress resistance of agricultural crops [18,19]. Chemical elicitors are stress factors that induce defense genes, leading to the accumulation of protective substances [20,21]. Elicitation is frequently used to promote the biosynthesis of valuable phytochemicals in callus cultures [22]. Salicylic acid (SA), methyl jasmonate (MeJA), and nitric oxide (NO) released by sodium nitroprusside (SNP) were shown to positively affect metabolic pathways, including CQAs biosynthesis [23]. For example, SA and MeJA improved CQAs accumulation in cell and hairy root cultures of *Gardenia jasminoides*, *Aster scaber*, *Mentha spicata*, and *Lactuca indica* [24,25,26]. In terms of the benefits to humanity, these compounds exhibit many health-promoting activities, such as antioxidant, antibacterial, antiobesity, antiviral, antidiabetic, antimicrobial, hypolipidemic, and antihypertension action [27,28,29,30,31]. Chlorogenic acid (5-CQA) is asserted to be the major compound among the CQAs in plants. It plays a significant role in the plant’s response to stress as an essential biosynthetic intermediate and exhibits several health-beneficial properties along with low toxicity and side effects [31,32,33].

*I. batatas* has recently received a lot of attention among researchers and society as another naturally transgenic plant and the first to be extensively used as food and feed for centuries [34]. Sweet potato genome carries two separate cellular T-DNA (cT-DNA) regions (*Ib*T-DNA1 and *Ib*T-DNA2). The foreign genes found in them are expressed at detectable levels in various plant tissues [34,35]. Especially, *Ib*T-DNA2 contains five ORFs homologous to the *Agrobacterium rhizogenes* T-DNA, namely open reading frame (ORF)13, ORF14, ORF17n, ORF18/ORF17n, and rooting locus (Rol)B/RolC proteins. These ORFs share sequence similarity with *plast* proteins (for phenotypic plasticity) recognized for their ability to cause pleiotropic effects in transformed plants and cell cultures [36,37,38]. Unfortunately, presently there is insufficient information on *Ib*T-DNA2 genes’ function in the physiological processes of sweet potatoes.

Plant cell cultures are regarded as a potential alternative for producing valuable natural compounds particularly in regions where plants cannot be cultivated regularly with abundant crops. Plant cell culture systems can also perform as models for studying the mechanisms of secondary metabolism regulation and stress responses, as the experiments could be replicated in the same conditions and without any external influences [21,39,40,41,42]. In comparison to whole plant cultivation, the callus culture technique provides some advantages, such as independence of climate or soil properties, the absence of the effect of insects and microorganisms, and others [43]. Traditional biotechnological techniques, like diverse elicitor supplementation to the medium, or genetic/genomic transformation, can effectively be implemented to enhance secondary metabolite production by cell cultures [20,44,45,46,47]. There are several reports on the establishment of callus cultures from *I. batatas* plants, in which phenolic compounds accumulation was studied [48,49,50,51,52]. However, sweet potato calli have never been studied with regard to the interplay of secondary metabolite biosynthesis, stresses, and cT-DNA gene expression.

This study aims to evaluate the effects of abiotic stresses (temperature, ultraviolet, and light) and chemical elicitors (methyl jasmonate, salicylic acid, and sodium nitroprusside) on the growth and accumulation of CQAs in *I. batatas* callus cultures as a model system. To justify the metabolic fluctuations, the study also looked into the expression patterns of the major biosynthetic genes under the studied experimental conditions. Furthermore, for the first time, expressional levels of the *Ib*T-DNA2 genes and the effect of *Ib-rolB/C* overexpression were examined using *I. batatas* cell culture.

## 2. Results and Discussion

### 2.1. Biomass Accumulation of I. batatas Calli under Stress Factors and Chemical Elicitor Treatment

**Figure 1:**
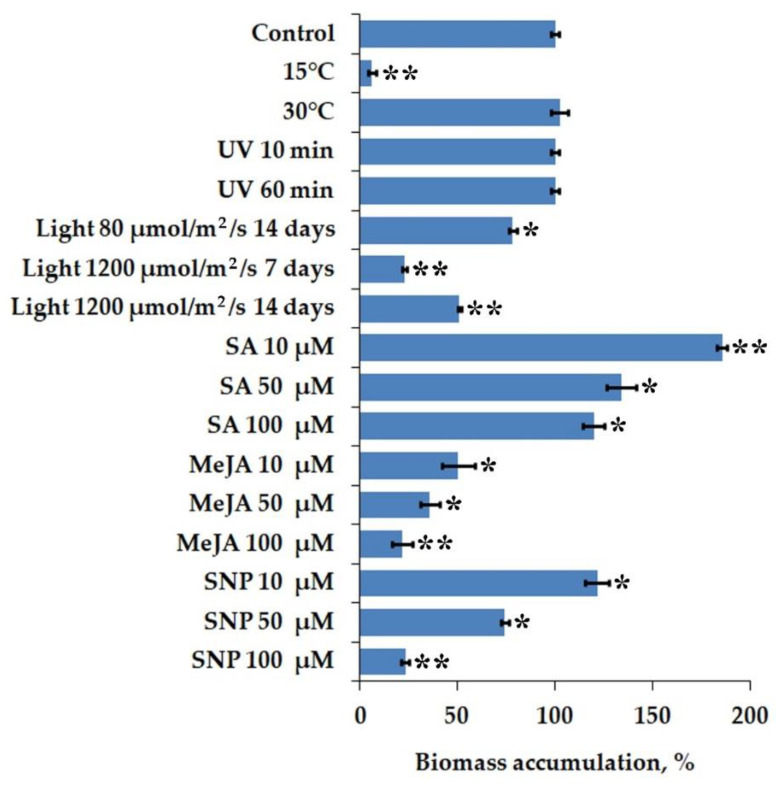
Accumulation of fresh biomass in *I. batatas* calli under abiotic stress and elicitor treatment. MeJA, methyl jasmonate; SA, salicylic acid; SNP, sodium nitroprusside. The data presented are mean values ± SE, * *p* < 0.05, ** *p* < 0.01.

We first aimed to evaluate the effect of abiotic stresses and chemical elicitors on biomass accumulation in *I. batatas* cell culture. We performed parallel cultivation under higher (30 °C and 37 °C) and lower (4 °C and 15 °C) temperatures (compared to normal conditions at 24 °C) to assess the temperature sensitivity. Severe temperature conditions (4 °C and 37 °C) almost entirely abolished callus growth; thus, these cells were not incorporated in the analysis. A temperature rise of 6 °C over control conditions displayed no significant effect on callus growth compared to normal conditions (Figure 1). Calli grown under cold (15 °C) conditions, despite being inhibited by about 90%, retained the growth ability. Consequently, sweet potato cultured cells demonstrated tolerance to higher temperatures while being sensitive to cold. A similar growth reduction under cold stress (13 °C) was exhibited in the callus of a legume *Cyclopia subternata* [53]. A higher incubation temperature changed *Oryza sativa* callus morphology but did not impact the growth [54]. Conversely, a notable rise in biomass accumulation at 30 °C was reported for *Solanum nigrum* callus culture [55]. In contrast to sweet potato cells, *Arabidopsis thaliana* and *Rubia cordifolia* calli were much more sensitive to a temperature rise of 4 °C over control conditions, while cold stress prevented their growth by only 72 and 81%, respectively [56].

Light is a crucial factor for plant growth and differentiation, capable of causing morphogenetic responses in cultured cells [57]. However, the sudden increase in light intensity, i.e., high light stress, leads to excessive reactive oxygen species (ROS) production and cell damage [58]. We studied the effect of normal (80 µmol/m^2^/s) and intense light (1200 µmol/m^2^/s) on the growth of *I. batatas* cell culture cultivated with a 16/8 h light/dark cycle (Figure 1). Normal light intensity reduced callus growth by 22% over control conditions (i.e., callus grown in darkness). The intense light exposure for 7 and 14 days remarkably inhibited cell growth by 77% and 49%, respectively, in comparison with that incubated in the dark. The latter outcome is quite unsuspected and may imply that prolonged light stress induced protective defense mechanisms in callus cells, enabling them to restore redox balance and recover biomass accumulation. Irradiation exposure of 30-day-old calli to UV-C light was conducted for 10 and 60 min and did not affect fresh weight levels (Figure 1). Ali et al. [59] observed that normal light intensity (40 µmol/m^2^/s) inhibited the *Artemisia absinthium* suspension cultures’ growth by 19%. Likewise, light reduced biomass accumulation of *O. sativa* cell culture [54]. Light conditions resulted in necrosis or browning in *Phoenix dactylifera* callus culture and this effect corresponded with the illumination intensity [57].

Chemical elicitors influenced the growth of *I. batatas* cell culture in a dose-dependent manner (Figure 1). Particularly, all doses of methyl jasmonate (MeJA) inhibited callus growth by 50–79% compared to the control. Conversely, 10 µM of salicylic acid (SA) promoted biomass accumulation significantly, while calli growth in the presence of 50 and 100 µM of SA remained unchanged. Low doses of sodium nitroprusside (SNP) (10 µM) demonstrated an apparent growth-promoting effect, while high concentrations of this elicitor (50 and 100 µM) caused a negative effect. Our results are in accordance with the previous reports [60,61,62], where lower concentrations of SA and SNP were able to induce biomass accumulation in plant cultures like *Fagonia indica*, *Allium sativum*, and *Gymnema sylvestre*. The effect of MeJA also was not uncommon as many investigators have previously demonstrated its inhibitory effects on plant cell growth [16,63,64,65].

### 2.2. Accumulation of Phytochemicals in I. batatas Calli under Various Stimuli

**Figure 2:**
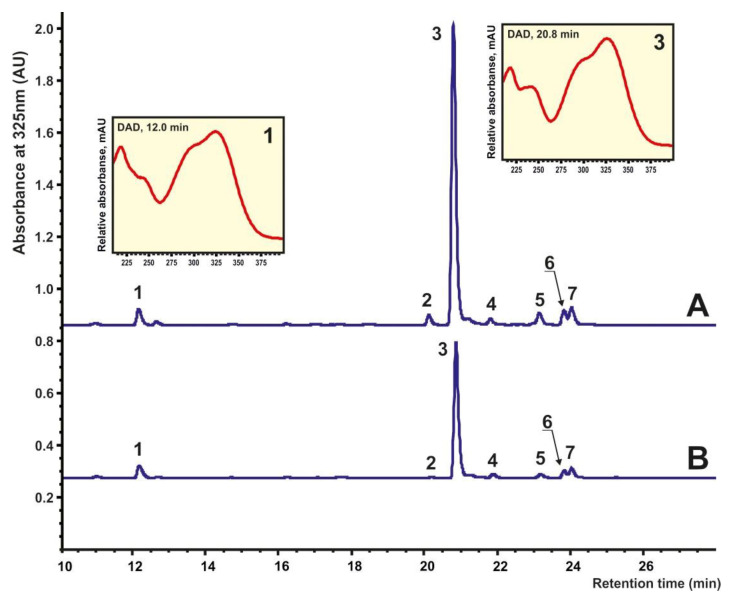
The HPLC-UV separation of main quinic acid derivatives from 3-day-old (**A**) and 30-day-old (**B**) *I. batatas* calli extracts, depicting the comparison of UV absorption profiles recorded at 325 nm. UV profiles of main compounds **1** (5-*O*-caffeoylquinic acid) and **3** (3,5-di-*O*-caffeoylquinic acid) recorded by DAD are presented.

*I. batatas* cell culture samples were collected from the 30-day-old calli for secondary metabolite extraction. Seven phenolic compounds were determined and identified using HPLC analysis of calli extracts (Figure 2). The characteristic maximum for all compounds at nearly 325 nm on their UV–vis spectra facilitated in defining them as phenolic acids. Compound **1** was identified as 5-CQA (5-*O*-caffeoylquinic acid) also known as chlorogenic acid (CGA) because of total compliance with the standard. Other compounds were identified using a published algorithm of the comparison of MS^n^ fragmentation patterns regarding sequences of chromatographic separation with the reverse phase column [66,67]. The monoisotopic molecular mass values were obtained using the HRMS with a mass error of below 4 ppm. As required, the multistage MS studies were performed. The chromatographic and mass-spectrometric data for all defined compounds needed for identification are summarized in Table S1. Consequently, six diacyl derivatives of quinic acid were found, with 3,5-dicaffeoylquinic acid (**3**) predominating in all samples. Other determined metabolites included 3,4-diCQA (3,4-di-*O*-caffeoylquinic acid) (**2**), 4,5-di-*O*-caffeoylquinic acid (4,5-diCQA) (**4**), 3-*O*-caffeoyl-5-*O*-coumaroylquinic acid (3C-5CoQA) (**5**), 3-*O*-feruloyl-5-*O*-caffeoylquinic acid (3F-5CQA) (**6**), and 3-*O*-caffeoyl-5-*O*-feruloylquinic acid (3C-5FQA) (**7**) (Figure 2, Table S1).

The effect of abiotic stress signals and chemical elicitors on the level of polyphenolic compounds in the cell culture of *I. batatas* is given in Table 1 and Table S2. Abiotic stresses induce contrasting effects on CQAs content in *I. batatas* calli. For example, CQAs concentration decreased by 2–5 times in low temperature and light conditions. However, higher growth temperature and UV-C irradiation caused up to 1.6-fold increase in CQAs content. As the later stimuli did not influence biomass accumulation significantly, the overall polyphenol production was increased to the same extent (Table 1 and Table S3).

Plants commonly activate secondary metabolite biosynthesis as a crucial part of their defense system to tackle stressful abiotic conditions [68]. Especially, phenolic compounds with antioxidant activity could mitigate the negative effect of ROS production with regard to stress stimuli [69,70]. However, the impact of stress signals on different plant species is not uniform. For example, in callus cultures of *Galega officinalis*, low temperature and UV- C irradiation improved secondary metabolite accumulation, while higher temperatures negatively impacted the phenolic acid levels [71]. Different light intensities noticeably stimulated the biosynthesis of secondary metabolites in cell cultures of *A. absinthium* and *P. dactylifera* [57,59], but this effect was not seen in our experiments with sweet potato cell culture.

Chemical elicitors, such as MeJA, SA, and SNP, are often used in plant cell cultures to enhance the biosynthesis of secondary metabolites [72,73]. Elicitation of callus cultures of *I. batatas* with different concentrations of SA, MeJA, and SNP exhibited a varying effect on secondary metabolite accumulation (Table 1). Cultures added with MeJA displayed a maximum elicitation of total CQAs content (18.32 mg/g DW) and (13.66 mg/g DW) at 50 and 100 µM of MeJA, respectively, as opposed to the control cells (4.55 mg/g DW). The maximum level for CQAs content (11.59 mg/g DW) was recorded at 10 µM of SA, while further growth in SA caused a much lower effect. Moreover, for cultures treated with SNP, the maximum value for total CQAs accumulation (7.57 mg/g DW) was recorded at 10 µM, and higher elicitor concentrations produced a two-fold decrease in secondary metabolite content. As opposed to the control callus culture, the highest production of CQAs (315.83 μg/L) was acquired from 10 μM of SA (Table 1 and Table S3).

In a similar study on hairy root cultures of *Eclipta prostrata*, MeJA greatly improved the CQAs production [74]. A growth in CGA and its derivatives in response to MeJA and SA in *Gardenia jasminoides* cell suspensions was also observed [24], although no effect of MeJA or SA on the biosynthesis of diCQAs was detected in *Cynara cardunculus* intact leaves [75]. SNP is a nitric oxide (NO) donor which causes secondary metabolism in the cell cultures of *G. sylvestre* [62], *A. annua* [76], and *Taxus yunnanensis* [77].

### 2.3. Expression Pattern of Biosynthetic Genes in I. batatas Calli

**Figure 3:**
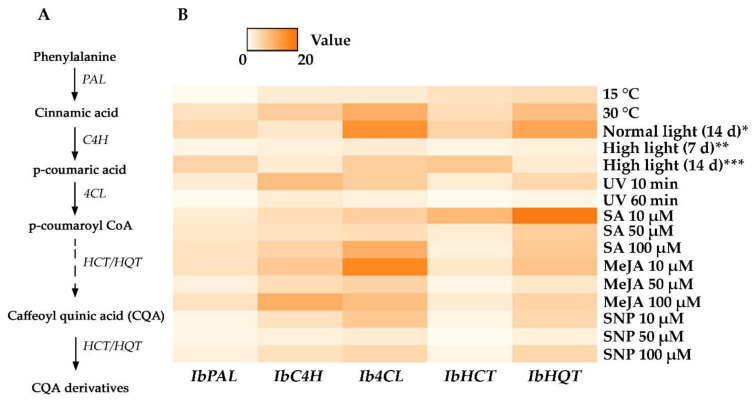
(**A**) Simplified representation of phenylpropanoid biosynthesis in *I. batatas*. PAL, L-phenylalanine ammonia-lyase; C4H, cinnamate 4-hydroxylase; 4CL, 4-coumarate-CoA ligase; HCT/HQT, 4-hydroxycinnamoyl-CoA—shikimate/quinate hydroxycinnamoyl transferase. The dotted arrow indicates several transformation steps. (**B**) A heatmap of the expression fold changes of *IbPAL*, *IbC4H*, *Ib4CL*, *IbHCT*, and *IbHQT* genes in *I. batatas* calli under abiotic stress growth conditions, relative to their expression levels in control callus. * 80 µmol/m^2^/s for 14 days, ** 1200 µmol/m^2^/s for 7 days, *** 1200 µmol/m^2^/s for 14 days. MeJA, methyl jasmonate; SA, salicylic acid; SNP, sodium nitroprusside.

CQAs biosynthesis has been proposed to occur through three alternative routes, and a total of 56 potential biosynthetic genes and 42 regulatory transcription factors have been recognized based on transcriptome profiling [10]. Among them, phenylalanine ammonia-lyase (PAL), cinnamate 4-hydroxylase (C4H), 4-coumarate CoA ligase (4CL), and 4-hydroxycinnamoyl-CoA—shikimate/quinate hydroxycinnamoyl transferase (HCT/HQT) play critical roles in phenylpropanoid pathway [78,79] (Figure 3A). We evaluated the changes in these gene expression patterns in *I. batatas* calli following abiotic stress and elicitor treatment by implementing qPCR analysis (Figure 3B).

Out of all the abiotic stress treatments, only high temperature, normal light, and UV-C irradiation considerably affected transcriptional levels of the studied biosynthetic genes. Specifically, *IbC4H*, *Ib4CL*, and *IbHQT* expression grew by 7-, 11-, and 9-fold, respectively, with regard to higher growth temperature (Figure 3B). UV-C treatment activated the expression of *IbC4H*, *Ib4CL*, and *IbHQT* genes 5–9 times more than the control. There was also notable growth in the expression of *Ib4CL* and *IbHQT* after low light exposure, yet this effect was not complementary to the CQAs content increase in treated calli. Feeding with chemical elicitors significantly improved the *IbC4H*, *Ib4CL*, and *IbHQT* expression, whereas the *IbPAL* expression did not change noticeably (Figure 3B). The maximum level for *IbC4H* expression (11-fold compared with control) was recorded at 100 µM of MeJA. The *Ib4CL* gene expression increased exceptionally by 11- and 17-fold after the 100 µM of MeJA and 10 µM of SA treatment, respectively. The highest mRNA abundance of *IbHQT* (19-fold compared with control) was acquired from 10 μM of SA. This treatment also induced the expression of the *IbHCT* gene 10 times more than in untreated cells. Among all chemical treatments, 10 µM of SA and MeJA had the most positive impact on the biosynthetic gene expression (Figure 3B). In general, it can be observed that the transcriptional activity of the upstream biosynthetic gene (*PAL*) was relatively lesser than those of downstream genes (*C4H*, *4CL*, and *HCT*/*HQT*) among all tested stress conditions (Figure 3B). A similar trend was noticed for equivalent phenylpropanoid biosynthetic gene expression in plantlets of *Agastache rugosa* subjected to LED illumination [80]. Han et al. [81] also found that the high levels of CQAs accumulation in *Iris germanica* rhizomes positively corresponded with the transcriptional activation of *C4H*, *4CL*, and *HQT*/*HCT* genes. Besides, RNAi-mediated suppression of *HQT* expression caused an almost complete inhibition of CGA biosynthesis in potatoes [27]. Moreover, the *C4H* and *HCT* genes displayed high expression levels under abiotic stress conditions in many plant species [70]. The transcriptional levels of *PAL* and *4CL* corresponded with the phenylpropanoid derivatives in *Melissa officinalis* cell culture [82]. An analogous trend was observed for *4CL* gene expression in elicitor-treated suspension cultures of *Echinacea purpurea* [83].

### 2.4. Analysis of IbT-DNA2 Genes Expression in I. batatas Calli

It is known that the naturally-transgenic *I. batatas* genome carries two independent cT-DNA copies, supporting at least nine intact open reading frames (ORFs) homologous to *Agrobacterium* spp. genes [34]. Especially, *Ib*T-DNA2 contains ORF13, ORF14, RolB/RolC homolog, ORF17n, and ORF18/ORF17n similar to those in *A. rhizogenes*. As the transformation of medical plants with wild-type *A. rhizogenes* is widely used for high-productive hairy root culture induction [84,85,86,87], we conducted the qPCR analysis of *ORF13*, *ORF14*, *Ib-rolB/C*, *ORF17n*, and *ORF18/17n* gene expression in *I. batatas* calli under control conditions and with reference to the studied stimuli.

Surprisingly, none of the *Ib*T-DNA2 genes was expressed in the cell culture of *I. batatas* under normal growth conditions (Table 2). Cold stress caused a weak up-regulation of *ORF13* but did not induce transcriptional levels of the remaining ORFs. Under normal light (80 µmol/m^2^/s) and UV-C treatment, the *Ib-rolB/C* gene expression considerably improved, with the most notable effect observed for low light intensity. The studied chemical elicitors did not affect the mRNA transcript abundance of *Ib*T-DNA2 genes in sweet potato calli. Consequently, the results imply that in the cell cultures of *I. batatas*, the expression of *Ib*T-DNA2 genes is not correlated with stress- and elicitor-induced regulation of secondary metabolism.

The silence of *Ib*T-DNA2 genes in the callus culture of *I. batatas* is currently uncertain. Previously it was found that *Ib-rolB/C* and *ORF13* were differently expressed in the apex, leaf, stem, tuber, and root tissues of *I. batatas* [34]. The expression of *NgrolB*, *NgrolC*, and *NgORF13*, cT-DNA genes in *Nicotiana glauca*, significantly increased in tobacco genetic tumors with regard to aging or wounding [88,89]. Callus culture acquired from *N. glauca* also carried the transcripts of these genes [90]. Additionally, our previous data imply that *rol* genes were transcriptionally active under their native promoters’ control when heterologously expressed in *R. cordifolia* transgenic calli [91,92]. It was observed that during prolonged cultivations, the transgene expression levels could reduce due to DNA methylation events [93]. The genetic instability of plant cells and tissue cultures is also well known [94,95]. However, sweet potato calli used in this study were approximately two-years-old, and *Ib*T-DNA2 transcripts were also undetectable in cDNA samples isolated in 2020 and 2021. It is possible that these genes are involved in processes, such as specific cellular differentiation, flowering, photosynthesis, and others, which are missing in cultured plant cells. For example, recent results showed that *Ib-rolB/C* gene promotes early flowering and premature leaf senescence in transgenic *Arabidopsis thaliana* plants [96].

### 2.5. Effect of Ib-rolB/C Gene Expression on Secondary Metabolism of Transgenic I. batatas Calli

**Figure 4:**
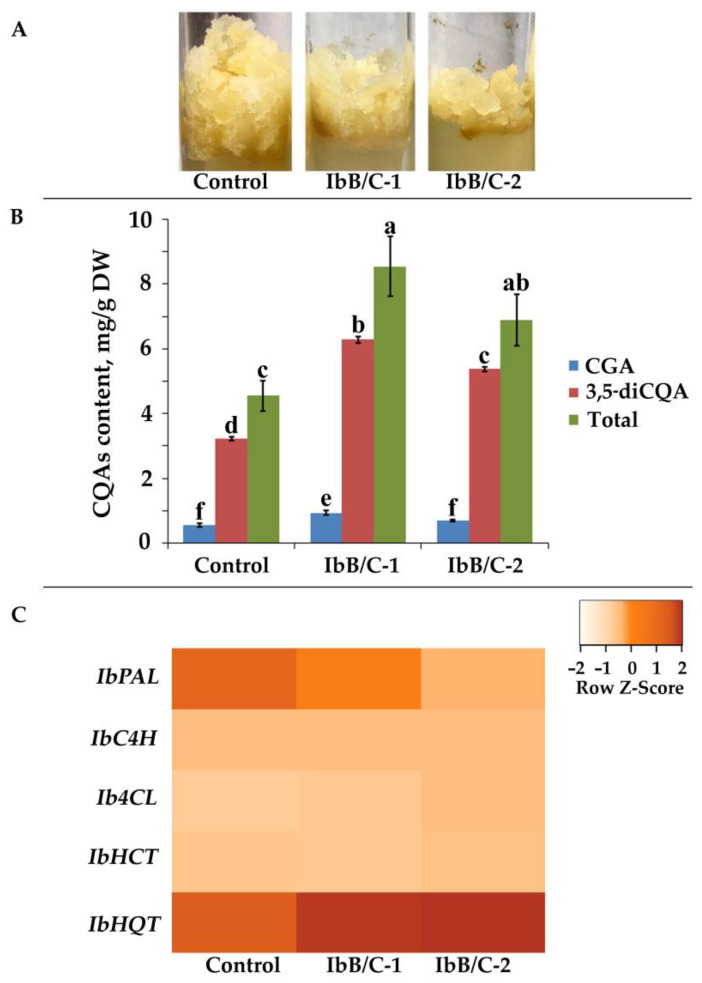
Characterization of *Ib-rolB/C*-expressing cell cultures of *I. batatas*. (**A**) The morphology of callus lines used in experiments. (**B**) The content of caffeoylquinic acids in control and transgenic calli. (**C**) A heatmap of the expression fold changes of *IbPAL*, *IbC4H*, *Ib4CL*, *IbHCT*, and *IbHQT* genes in control and transgenic calli. The data presented are mean values ± SE. Different letters above the bars indicate statistically significant differences in means (*p* < 0.05), Fisher’s LSD.

The callus culture of *I. batatas* was transformed with *A. tumefaciens* strain harboring the pPZP-RCS2-Ocs:nptII/35S:rolBC construct to establish transgenic cell lines overexpressing the full-length *Ib-rolB/C* gene. PCR established the successful transfer of exogenous T-DNA with *nptII*-specific primers after the 6-month selection of transformed callus aggregates on a 50 mg/L kanamycin-carrying medium. The *Ib-rolBC*-transgenic cell lines represented moderately growing homogenous calli, which did not exhibit signs of differentiation and displayed similar phenotypes with their parental cell line (Figure 4A). For further investigation, we used an untransformed callus as control and two independently obtained transgenic cell lines, namely IbB/C-1 and IbB/C-2. The RolB/RolC coding sequence in pPZP-RCS2-Ocs:nptII/35S:rolBC binary vector was used to differentiate between transgenic and native *Ib-rolB/C* genes with a special forward primer corresponding to tobacco etch virus 5′-UTR situated upstream (Figure S1). IbB/C-1 and IbB/C-2 lines expressed the transgenic *Ib-rolB/C* gene in the same levels, while no endogenous *Ib-rolB/C* or other *Ib*T-DNA2 gene expression was detected using qPCR. Besides, overexpression of *Ib-rolB/C* gene reduced biomass accumulation of IbB/C-1 and IbB/C-2 calli by 1.2- and 1.6-times, respectively (Table S4). A similar growth reduction was exhibited in several plant cell cultures overexpressing *rolB* gene from the wild-type *A. rhizogenes* strain A4 [36,39,92].

Metabolite profiling disclosed the impact of *Ib-rolB/C* transgene on different phenylpropanoids (Figure 4B; Table S4). Particularly, CGA grew approximately 1.7-fold, whereas 3,5-diCQA increased 1.7- to 2.0-fold in transgenic cell lines. The highest total content and production of CQAs were acquired in the IbB/C-1 line and reached 8.55 mg/g DW and 106.32 mg/L (Figure 4B; Table S4), which is 1.9- and 1.6-times higher than the control cells, respectively.

To verify the increased production of CQAs in the transgenic cell lines, we examined the expression levels of biosynthetic genes (Figure 4C). The obtained data demonstrated that the overexpression of the *Ib-rolB/C* gene in transgenic cell lines reduced the *IbPAL* transcript levels in transgenic cell lines, with the corresponding expression levels being 1.5–4.9 times lower than the control. A possible justification for this effect could be that the increased amounts of CQAs in transgenic calli contributed to the regulation of *IbPAL* transcription. Previously Payyavula et al. [27] proposed the existence of such interconnection in the regulation of CGA biosynthesis in potatoes. On the contrary, we found a considerable increase in the transcript levels of the *IbHQT* by approximately 2-times (Figure 4C). Therefore, our data suggest that CQAs are primarily synthesized through the HQT pathway in *Ib-rolB/C*-expressing cell cultures of *I. batatas*. The pivotal role of HQT in the biosynthesis of CQAs was previously reported for several plant species [97,98,99].

## 3. Materials and Methods

### 3.1. Plant Cell Cultures and Treatments

Tubers of sweet potato *I. batatas* were purchased from the local supplier and germinated in well-drained sandy soil (pH 6–6.5) under a 16 h light/8 h dark photoperiod at 25 °C. Leaves of the 40-day-old sweet potato plantlets were used for callus induction. For sterilization, leaf segments were immersed in 1% mercuric chloride for 30 s followed by washing three times with autoclaved distilled water. Sanitized explants were transferred to Murashige and Skoog (MS) [100] medium supplemented with plant growth regulator 4-chlorophenoxyacetic acid (4-CPA) (0.5 mg/L). Routine cultivation of *I. batatas* calli was conducted in the dark at 24 °C with 30-day subculture intervals. For abiotic stress experiments, which involved temperature and light stress, 150 mg of calli were placed in 20 × 200 mm glass test tubes containing 15 mL of medium. A temperature stress was performed with calli cultivated at 4, 15, 30, and 37 °C for the entire subculture period in a KS-200 climatic test chamber (Smolensk SKTB SPU, Smolensk, Russia). The ultraviolet treatments at 254 nm wavelength (UV-C) were conducted using 30-day-old calli for 10 or 60 min using a UV lamp R-52G (UVP Inc., San Gabriel, CA, USA). Additionally, the 16-day-old calli were subjected to normal light at 80 µmol/m^2^/s for 14 days and intense light at 1200 µmol/m^2^/s for 7 and 14 days under a 16 h light/8 h dark photoperiod. Chemical elicitors, salicylic acid (SA), methyl jasmonate (MeJA), and sodium nitroprusside (SNP), were added to the medium aseptically at concentrations of 0, 10, 50, and 100 μM, respectively, afterwards calli were grown under the usual conditions. At the end of the experiments, samples were harvested, weighed, and used for chemical and gene expression analysis.

### 3.2. High-Performance Liquid Chromatography (HPLC) Analysis

For secondary metabolite analysis *I. batatas* calli were dried under hot air for 20 h and ground using mortar and pestle.

#### 3.2.1. Chemicals

Analytical standards (chlorogenic acid and cynarin) were obtained from Sigma-Aldrich (St. Louis, MI, USA). All extraction solutions and eluents were prepared with ultra-pure water (Millipore, Burlington, MA, USA). All solvents were of analytical grade.

#### 3.2.2. Sample Preparation for Analytical Chromatography

The oven-dried (at 50 °C in the darkness to a constant weight) and powdered plant material was homogenized with two volumes (*w/v*) of 80% aqueous methanol. Then, the homogenates were sonicated at 40 °C for 30 min, equilibrated for 10 h in the darkness, and centrifuged (15,000× *g*, 15 min). The supernatant was filtered and the residue was re-extracted once more in the same manner. The extracts were combined and cleared with a 0.45-μm membrane (Millipore, Burlington, MA, USA) and used for HPLC analysis.

#### 3.2.3. Analytical Chromatography and Mass-Spectrometry

The analytical chromatography was performed at the Instrumental Centre of Biotechnology and Gene Engineering of FSC Biodiversity FEB RAS using a 1260 Infinity analytical HPLC system (Agilent Technologies, Santa Clara, CA, USA), equipped with a photodiode array detector (DAD). An analytical Zorbax C18 column (150 mm, 2.1-mm i.d., 3.5-μm part size, Agilent Technologies, Santa Clara, CA, USA) was applied for separation. The column temperature was supported at 40 °C. The mobile phase consisted of a gradient elution of 0.1% aqueous formic acid (A) and acetonitrile (B). The gradient profile with a flow rate of 0.2 mL/min was: 0 min 5% B; 20 min 30% B; 30 min 100% B, and then eluent B until 40 min. The injection volume was 1–5 μL. UV spectra were recorded with a DAD in the range between 200 and 400 nm. Chromatograms for quantification were obtained at a wavelength of 325 nm. Instrument operation, data collection, and analysis were controlled using the Agilent OpenLAB CDS software (v.01.06.111).

The HPLC system was interfaced with an ion trap mass spectrometer Bruker HCT ultra PTM Discovery System (Bruker Daltonik GmbH, Bremen, Germany) equipped with an electrospray ionization (ESI) source. The low-resolution MS investigations were carried out in negative ion detection. The following settings were used: the range of *m/z* detection was 100–1000, the drying gas (N_2_) flow rate was 8.0 L/min, the nebulizer gas (N_2_) pressure was 25 psi, the ion source potential was 3.8 kV. and the drying gas temperature was 325 °C. Tandem mass spectra were acquired in Auto-MS^2^ mode (smart fragmentation) using a ramping of the collision energy. The fragmentation amplitude was set to 1 V. As required, MS^3^ experiments were performed. MS data were collected using the Bruker Daltonics Compass 1.3 esquire control software (v.6.2.581.3) and processed with the Bruker Daltonics Compass 1.3 Data Analysis software (v.4.0.234.0).

The high-resolution MS spectra were recorded using a Shimadzu LCMS-IT-TOF instrument (Shimadzu, Kyoto, Japan) including LC-20AD Prominence and tandem ion-trap/time-of-flight mass spectrometer. The mass spectra were collected applying ESI conditions with simultaneous negative and positive ion detection with a mass resolution up to 12,000. The following settings were used: the range of *m/z* detection was 100–1000, the drying gas (N_2_) pressure was 200 kPa, the nebulizer gas flow rate was 1.5 L/min, the ion source potential changed from −3.8 to 4.5 kV, and the interface temperature was 200 °C. MS data were collected and processed using the Shimadzu LC-MS Solution software (v.3.60.361).

All identified quinic acid derivatives were quantified using HPLC with DAD detection at a wavelength of 325 nm on the base of four-point regression curves built with the reference standards. Two commercial standards of the quinic acid derivatives were available in our laboratory: chlorogenic acid and cynarin. Cynarin was used for quantification of diacyl quinic acid derivatives.

### 3.3. Obtaining Ib-rolB/C–Expressing Cell Cultures

The full-length sequence of the *Ib-rolB/C* gene (Genbank Acc. no. KM052617, 5158–5868 bp) was PCR-amplified from *I. batatas* DNA and cloned into pPZP-RCS2-Ocs:nptII binary vector [101] as described earlier [96]. Construct pPZP-RCS2-Ocs:nptII/35S:rolBC carries *Ib-rolB/C* gene under the control of cauliflower mosaic virus (CaMV) 35S promoter and encodes the neomycin phosphotransferase (*nptII*) gene for kanamycin selection. The RolB/RolC coding sequence in pPZP-RCS2-Ocs:nptII/35S:rolBC binary vector also contains the translational enhancer element derived from the tobacco etch virus 5′-untranslated region (5′-UTR) situated upstream. The obtained vector was checked for the absence of mutations by DNA sequencing using an ABI 3500 Genetic Analyzer (Applied Biosystems, Foster City, CA, USA) as described earlier [102] and transferred into *Agrobacterium tumefaciens* strain EHA105/pTiBo542 [103] using a GenePulser Xcell electroporation system (Bio-Rad Laboratories, Inc., Hercules, CA, USA) in accordance with the manufacturer’s protocol.

The transformed *Ib-rolB/C*-transgenic callus lines of *I. batatas* were obtained using *A. tumefaciens* strain EHA105 harboring the pPZP-RCS2-Ocs:nptII/35S:rolBC construct from the control cell culture as described earlier [104]. Several *Ib-rolB/C*-expressing kanamycin-resistant cell lines were selected from independently transformed callus aggregates and two of them, designated as IbB/C-1 and IbB/C-2, were used in this study. Using conventional PCR analyses with *nptII*-specific primers [102,104], we confirmed T-DNA integration in the two transgenic cell lines. The transformed *Ib-rolB/C*-expressing callus lines were cultivated under the same conditions as untransformed control calli.

### 3.4. Synthesis of cDNA and Real-Time PCR Analysis

Total RNA was isolated from 150 mg of callus tissue using the Lira reagent kit (Biolabmix, Novosibirsk, Russia) according to the manufacturer’s protocol. First-strand cDNA synthesis and quantitative real-time PCR (qPCR) analysis were performed as described previously [91] on a CFX96 thermal cycler (Bio-Rad Laboratories, Hercules, CA, USA) with 2× BioMaster HS-qPCR SYBR Blue (Biolabmix, Russia).

The gene-specific primer pairs used in the qPCR are listed in Table S5. Ubiquitin gene of *I. batatas* was used as reference gene. Primer pair efficiency of >95 % was verified with a standard curve that was established using serial dilutions of the corresponding purified PCR products. Three biological replicates, resulting from independent RNA extractions, were used and three technical replicates were analyzed for each biological replicate. Data were analyzed using CFX Manager Software ver. 3.1 (Bio-Rad Laboratories, Hercules, CA, USA).

### 3.5. Statistical Analysis

All values were expressed as the mean ± SE. For statistical evaluation, Student’s *t* test was used to compare the two independent groups. For comparison among multiple data, analysis of variance (ANOVA) followed by a multiple comparison procedure was employed. Fisher’s protected least significant difference (PLSD) post hoc test was employed for the inter-group comparison. The level of statistical significance was set at *p* < 0.05. Pearson correlation analysis was used to reveal relationships between two variables.

## 4. Conclusions

In this study, we examined the influence of selected abiotic stresses and chemical elicitors on the growth and secondary metabolite biosynthesis in cell cultures of *I. batatas*. It was observed that each of the treatments applied to the calli impacted the CQAs accumulation and production in different ways. Higher growth temperature and UV-C irradiation were the most efficient stress treatments for CQAs accumulation and production. The most effective elicitor treatment (SA) induced a considerable accumulation of higher amounts of phenolic secondary metabolites (5-fold increase) in the cell cultures of sweet potatoes. This can be implemented to enhance the potential of *I. batatas* plant-cell-based production of essential natural compounds. *Ib*T-DNA2 genes were not expressed in untreated sweet potato cell culture and stress signals only slightly influenced their transcriptional levels. Thus, these genes are not implicated in stress- and elicitor-induced regulation of the secondary metabolism of cultured *I. batatas* cells. However, overexpression of the *Ib-rolB/C* gene induced up to a 1.9-fold increase in the content of total CQAs. We propose that this result was obtained because of the activation of HQT, a downstream enzyme in the CQAs metabolic pathway, through as-yet-uncharacterized signaling pathways.

## Figures and Tables

**Table 1:** Content and production of CQAs in *I. batatas* calli under abiotic stress and elicitor treatment.

Callus Line	CQAs Content, mg/g DW			Dry Weight (g/L)	CQAs Production, mg/L		
	CGA	3,5-diCQA	Total CQAs		CGA	3,5-diCQA	Total CQAs
Control	0.56 ± 0.05	3.23 ± 0.08	4.55 ± 0.47	14.67 ± 0.29	8.23 ± 0.16	47.40 ± 0.95	66.78 ± 1.34
15 °C	0.14 ± 0.02	0.58 ± 0.08	0.98 ± 0.08	0.88 ± 0.28	0.13 ± 0.04	0.51 ± 0.16	0.86 ± 0.27
30 °C	1.10 ± 0.14	5.29 ± 0.74	7.34 ± 0.78	15.01 ± 0.62	16.58 ± 0.68	79.39 ± 3.27	110.14 ± 4.53
Normal light *	0.29 ± 0.04	1.99 ± 0.28	2.68 ± 0.29	11.47 ± 0.28	3.35 ± 0.09	22.83 ± 0.58	30.74 ± 0.79
High light (7 d) **	0.22 ± 0.03	1.40 ± 0.20	1.97 ± 0.20	3.34 ± 0.14	0.75 ± 0.03	4.68 ± 0.21	6.59 ± 0.29
High light (14 d) ***	0.25 ± 0.03	1.33 ± 0.19	1.90 ± 0.19	7.46 ± 0.15	1.87 ± 0.04	9.90 ± 0.19	14.16 ± 0.28
UV 10 min	0.81 ± 0.10	5.50 ± 0.77	7.33 ± 0.81	14.67 ± 0.29	11.84 ± 0.24	80.76 ± 1.62	107.51 ± 2.15
UV 60 min	0.52 ± 0.07	4.15 ± 0.58	5.41 ± 0.61	14.67 ± 0.29	7.64 ± 0.15	60.93 ± 1.22	79.42 ± 1.59
SA 10 μM	1.00 ± 0.13	8.59 ± 1.20	11.59 ± 1.25	27.25 ± 0.39	27.24 ± 0.39	234.08 ± 3.39	315.83 ± 4.58
SA 50 μM	0.85 ± 0.11	6.88 ± 0.96	9.35 ± 1.00	19.65 ± 1.10	16.74 ± 0.94	135.08 ± 7.54	183.76 ± 10.26
SA 100 μM	0.48 ± 0.06	3.60 ± 0.50	4.77 ± 0.53	17.57 ± 0.82	8.39 ± 0.39	63.34 ± 2.97	83.91 ± 3.94
MeJA 10 μM	0.85 ± 0.11	7.52 ± 1.05	9.85 ± 1.11	7.36 ± 1.23	6.24 ± 1.05	55.34 ± 9.28	72.51 ± 12.15
MeJA 50 μM	1.22 ± 0.16	14.47 ± 2.03	18.32 ± 2.14	5.24 ± 0.75	6.41 ± 0.92	75.82 ± 10.85	96.01 ± 13.74
MeJA 100 μM	0.91 ± 0.12	9.87 ± 1.38	13.66 ± 1.43	3.14 ± 0.78	2.86 ± 0.71	31.02 ± 7.72	42.95 ± 10.69
SNP 10 μM	0.76 ± 0.10	5.66 ± 0.79	7.57 ± 0.83	17.84 ± 0.90	13.47 ± 0.68	101.03 ± 5.08	135.11 ± 6.79
SNP 50 μM	0.38 ± 0.05	1.98 ± 0.28	2.69 ± 0.29	10.87 ± 0.30	4.10 ± 0.11	21.54 ± 0.59	29.28 ± 0.80
SNP 100 μM	0.38 ± 0.05	1.98 ± 0.28	2.65 ± 0.29	3.39 ± 0.31	1.29 ± 0.12	6.71 ± 0.62	8.96 ± 0.82
		↓ > 2	↓ < 2	1	↑ < 2	↑ > 2	

* 80 µmol/m^2^/s for 14 days, ** 1200 µmol/m^2^/s for 7 days, *** 1200 µmol/m^2^/s for 14 days. MeJA, methyl jasmonate; SA, salicylic acid; SNP, sodium nitroprusside. The data presented are mean values ± SE. Coloration of the cells indicates statistically significant differences of means (*p* < 0.05) in the columns, Fisher’s LSD. Color ranges according to the magnitude of CQAs content and production changes relative to control, as shown in the scale bar. *↓* and *↑* denote lower and higher levels, respectively.

**Table 2:** Expression of cT-DNA ORFs in *I. batatas* calli under abiotic stress and elicitor treatment.

	*Ib-rolB/C*	*ORF13*	*ORF14, ORF17n, ORF18/17n*
Control	UD	UD	UD
15 °C	UD	1.07 ± 0.08	UD
30 °C	UD	UD	UD
Normal light *	5.17 ± 0.12	UD	UD
High light **	UD	UD	UD
UV 10 min	2.49 ± 0.09	UD	UD
UV 60 min	3.21 ± 0.11	UD	UD
Chemical elicitors (MeJA, SA, and SNP)	UD	UD	UD

* 80 µmol/m^2^/s for 14 days, ** 1200 µmol/m^2^/s for 7 or 14 days. MeJA, methyl jasmonate; SA, salicylic acid; SNP, sodium nitroprusside. UD—undetectable levels. The data presented are mean values ± SE.

## Data Availability

The datasets generated during and/or analyzed during the current study are available from the corresponding author on reasonable request.

## Supplementary Materials

The supporting information can be downloaded at: https://www.mdpi.com/article/10.3390/ijms232315100/s1. References [105,106] are cited in the Supplementary Materials.
