# Supplementary material for: Effect of Stress Signals and Ib-rolB/C Overexpression on Secondary Metabolite Biosynthesis in Cell Cultures of Ipomoea batatas
Source: Int J Mol Sci. 2022 Dec 1;23(23):15100. doi: 10.3390/ijms232315100 (PMC9740395; doi:10.3390/ijms232315100)
Supplement: Supplementary file 1 [file ijms-23-15100-s001.zip › ijms-2033802-supplementary.pdf]

Supplementary Materials

Effect of stress signals and *Ib-rolB/C* overexpression on secondary metabolite biosynthesis in cell cultures of *Ipomoea batatas*

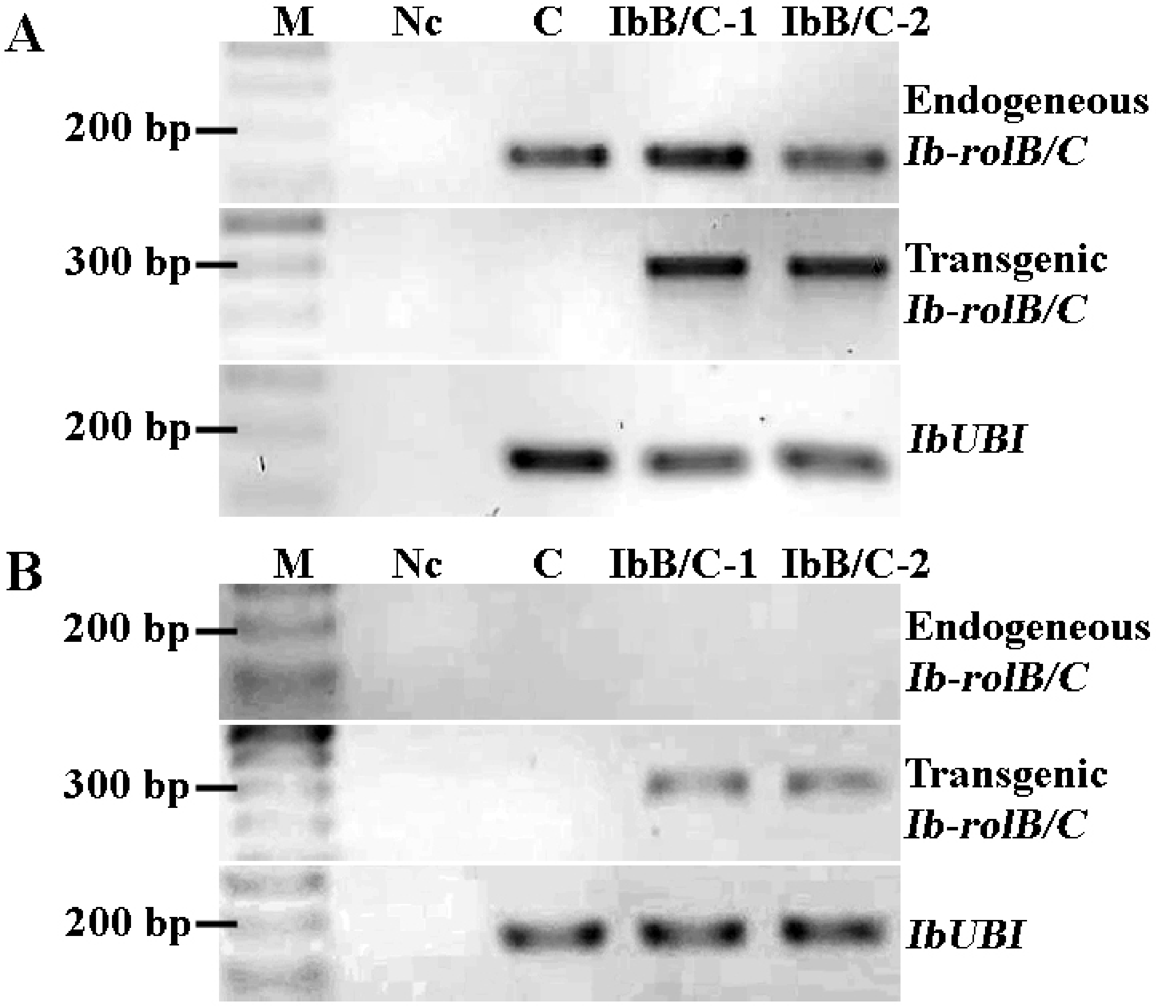

Figure S1. Detection of endogenous and transgenic *Ib-rolB/C* gene in *I. batatas* calli. (A) Genomic DNA and (B) cDNA PCR analysis from control and *Ib-rolB/C*-transgenic cell cultures of *I. batatas*. The amplified product for the endogenous *Ib-rolB/C* sequence was 137 bp, while transgenic sequence containing TEV 5'-UTR was 291 bp. C, control callus line; IbB/C-1 and IbB/C-2, *Ib-rolBC*-transgenic callus lines; M, 100 bp DNA ladder; NC, negative control (water).

Table S1. List of main CQAs identified in *I batatas* calli using HPLC-UV-ESI-MS(/MS²).

No	Rt (min)	UVmax (nm)	$[M–H]^-$ (*m/z*) detected	theoretical	Diff (ppm)	Molecular Formula	MS² fragmentation, main diagnostic ions (% of base peak) (*m/z*)	MS³ fragmentation, main diagnostic ions (% of base peak) (*m/z*)	Compound identification, or isomer	References
1	12.0	325	353.0868	353.0878	2.84	$C_{16}H_{18}O_9$	191(100); 179(5)		CGA (5-*O*-caffeoylquinic acid)	St, 1, 3
2	20.1	325	515.1187	515.1195	1.55	$C_{25}H_{24}O_{12}$	**353***(100); 335(17); 173(22)	191(27);179(50); 173(100)	3,4-diCQA (3,4-di-*O*-caffeoylquinic acid)	1, 3, 4
3	20.8	326	515.1177	515.1195	3.49	$C_{25}H_{24}O_{12}$	**353***(100); 191(7)	191(100); 179(66); 135(10)	3,5-diCQA (3,5-di-*O*-caffeoylquinic acid)	1, 3, 4
4	21.8	327	515..1178	515.1195	3.29	$C_{25}H_{24}O_{12}$	**353***(100); 173(12)	191(65); 179(55); 173(100)	4,5-diCQA (4,5-di-*O*-caffeoylquinic acid)	1, 3, 4
5	23.2	316	499.1235	499.1246	2.17	$C_{25}H_{24}O_{11}$	353(100); 337(11); 191(8)		3C-5CoQA (3-*O*-caffeoyl-5-*O*-coumaroylquinic acid)	2
6	23.8	326	529.1334	529.1352	3.30	$C_{26}H_{26}O_{12}$	367(100); 335(3);193(9)		3F-5CQA (3-*O*-feruloyl-5-*O*-caffeoylquinic acid)	1, 4
7	24.0	326	529.1331	529.1352	3.87	$C_{26}H_{26}O_{12}$	367(44); 353(100); 191(12)		3C-5FQA (3-*O*-caffeoyl-5-*O*-feruloylquinic acid)	1, 4

* – precursor ions for MS³ are accentuated in bold. St – standard sample

References for identification:

1. Clifford, M.N.; Johnston, K.L.; Knight, S.; Kuhnert, N. Hierarchical scheme for LC-MSⁿ identification of chlorogenic acids. *J. Agric. Food Chem*. **2003**, *51*, 2900-2911.
2. Clifford, M.N.; Marks, S.; Knight, S.; Kuhnert, N. Characterization by LC-MSⁿ of four new classes of *p*-coumaric acid - containing diacyl chlorogenic acids in green coffee beans. *J. Agric. Food Chem.* **2006**, *54*, 4095-4101.
3. Sasaki, K.; Oki, T.; Kobayashi, T.; Kai, Y.; Okuno, S. Single-laboratory validation for the determination of caffeic acid and seven caffeoylquinic acids in sweet potato leaves. *Biosci. Biotechnol. Biochem.* **2014**, *78*, 2073-2080.
4. Zhang, L.; Tu, Z.; Wang, H.; Fu, Z.; Wen, Q.; Chang, X.; Huang, X. Comparison of different methods for extracting polyphenols from *Ipomoea batatas* leaves, and identification of antioxidant constituents by HPLC-QTOF-MS². *Int. Food Res. J.* **2015**, *70*, 101–109.

Table S2. Content of CQAs in 30-days-old *I. batatas* calli under abiotic stresses and elicitors treatment.

Callus line	Dry weight (g/L)	CQAs content, mg/g DW								
		CGA	3,5-diCQA	3,4-diCQA	4,5-diCQA	3C-5CoQA	3F-5CQA	3C-5FQA	Total diCQAs	Total CQAs
Control	14.67±0.29	0.56±0.05	3.23±0.08	0.18±0.01	0.12±0.01	0.06±0.00	0.18±0.02	0.22±0.03	3.99±0.56	4.55±0.47
15˚C	0.88±0.28	0.14±0.02	0.58±0.08	0.05±0.01	0.02±0.00	0.03±0.00	0.12±0.01	0.03±0.00	0.83±0.10	0.98±0.08
30˚C	15.01±0.62	1.10±0.14	5.29±0.74	0.05±0.01	0.13±0.02	0.25±0.04	0.22±0.02	0.29±0.04	6.23±0.93	7.34±0.78
UV 10 min	14.67±0.29	0.81±0.10	5.50±0.77	0.18±0.04	0.26±0.03	0.10±0.01	0.19±0.02	0.28±0.04	6.52±0.97	7.33±0.81
UV 60 min	14.67±0.29	0.52±0.07	4.15±0.58	0.10±0.02	0.21±0.03	0.11±0.02	0.12±0.01	0.20±0.03	4.89±0.73	5.41±0.61
Normal light*	11.47±0.28	0.29±0.04	1.99±0.28	0.07±0.02	0.10±0.01	0.04±0.01	0.09±0.01	0.09±0.01	2.39±0.35	2.68±0.29
High light (7 d)**	3.34±0.14	0.22±0.03	1.40±0.20	0.09±0.02	0.10±0.01	0.03±0.00	0.07±0.01	0.07±0.01	1.75±0.24	1.97±0.20
High light (14 d)***	7.46±0.15	0.25±0.03	1.33±0.19	0.04±0.01	0.05±0.01	0.05±0.01	0.10±0.01	0.08±0.01	1.65±0.23	1.90±0.19
SA 10 µM	27.25±0.39	1.00±0.13	8.59±1.20	0.13±0.03	0.31±0.04	0.37±0.05	0.42±0.04	0.77±0.11	10.59±1.50	11.59±1.25
SA 50 µM	19.65±1.10	0.85±0.11	6.88±0.96	0.03±0.01	0.20±0.02	0.26±0.04	0.39±0.04	0.74±0.10	8.50±1.20	9.35±1.00
SA 100 µM	17.57±0.82	0.48±0.06	3.60±0.50	0.07±0.02	0.14±0.02	0.12±0.02	0.14±0.01	0.22±0.03	4.30±0.63	4.77±0.53
MeJA 10 µM	7.36±1.23	0.85±0.11	7.52±1.05	0.08±0.02	0.23±0.03	0.23±0.03	0.33±0.03	0.61±0.09	9.00±1.32	9.85±1.11
MeJA 50 µM	5.24±0.75	1.22±0.16	14.47±2.03	0.18±0.04	0.34±0.04	0.33±0.05	0.65±0.06	1.12±0.16	17.10±2.55	18.32±2.14
MeJA 100 µM	3.14±0.78	0.91±0.12	9.87±1.38	0.49±0.12	0.25±0.03	0.42±0.06	1.02±0.09	0.69±0.10	12.75±1.70	13.66±1.43
SNP 10 µM	17.84±0.90	0.76±0.10	5.66±0.79	0.10±0.02	0.31±0.04	0.15±0.02	0.22±0.02	0.38±0.05	6.82±0.99	7.57±0.83
SNP 50 µM	10.87±0.30	0.38±0.05	1.98±0.28	0.04±0.01	0.12±0.01	0.04±0.01	0.06±0.01	0.08±0.01	2.32±0.35	2.69±0.29
SNP 100 µM	3.39±0.31	0.38±0.05	1.98±0.28	0.02±0.01	0.07±0.01	0.05±0.01	0.06±0.01	0.09±0.01	2.26±0.35	2.65±0.29

* 80 µmol/m^{2}/s for 14 days, ** 1200 µmol/m^{2}/s for 7 days, *** 1200 µmol/m^{2}/s for 14 days

Table S3. Production of CQAs in in 30-days-old *I. batatas* calli under abiotic stresses and elicitors treatment.

	CQAs production, mg/L								
**Callus line**	**CGA**	**3,5-diCQA**	**3,4-diCQA**	**4,5-diCQA**	**3C-5CoQA**	**3F-5CQA**	**3C-5FQA**	**Total diCQAs**	**Total CQAs**
Control	8.23±0.16	47.40±0.95	2.64±0.05	1.81±0.04	0.95±0.02	2.59±0.05	3.16±0.06	58.55±1.17	66.78±1.34
15˚C	0.13±0.04	0.51±0.16	0.05±0.02	0.02±0.01	0.03±0.01	0.10±0.03	0.03±0.01	0.73±0.23	0.86±0.27
30˚C	16.58±0.68	79.39±3.27	0.81±0.03	1.95±0.08	3.82±0.16	3.25±0.13	4.36±0.18	93.57±3.85	110.14±4.53
UV 10 min	11.84±0.24	80.76±1.62	2.67±0.05	3.77±0.08	1.54±0.03	2.82±0.06	4.11±0.08	95.67±1.91	107.51±2.15
UV 60 min	7.64±0.15	60.93±1.22	1.49±0.03	3.06±0.06	1.62±0.03	1.73±0.03	2.93±0.06	71.78±1.44	79.42±1.59
Normal light*	3.35±0.09	22.83±0.58	0.85±0.02	1.16±0.03	0.45±0.01	1.02±0.03	1.08±0.03	27.39±0.70	30.74±0.79
High light (7 d)**	0.75±0.03	4.68±0.21	0.30±0.01	0.32±0.01	0.10±0.00	0.22±0.01	0.22±0.01	5.84±0.26	6.59±0.29
High light (14 d)***	1.87±0.04	9.90±0.19	0.33±0.01	0.36±0.01	0.36±0.01	0.72±0.01	0.62±0.01	12.29±0.24	14.16±0.28
SA 10 µM	27.24±0.39	234.08±3.39	3.43±0.05	8.51±0.12	10.15±0.15	11.35±0.16	21.06±0.31	288.59±4.18	315.83±4.58
SA 50 µM	16.74±0.94	135.08±7.54	0.61±0.03	3.98±0.22	5.20±0.29	7.68±0.43	14.47±0.81	167.02±9.33	183.76±10.26
SA 100 µM	8.39±0.39	63.34±2.97	1.31±0.06	2.54±0.12	2.08±0.10	2.39±0.11	3.85±0.18	75.51±3.54	83.91±3.94
MeJA 10 µM	6.24±1.05	55.34±9.28	0.56±0.09	1.71±0.29	1.70±0.28	2.47±0.41	4.49±0.75	66.26±11.11	72.51±12.15
MeJA 50 µM	6.41±0.92	75.82±10.85	0.96±0.14	1.80±0.26	1.74±0.25	3.42±0.49	5.87±0.84	89.60±12.82	96.01±13.74
MeJA 100 µM	2.86±0.71	31.02±7.72	1.55±0.39	0.79±0.20	1.34±0.33	3.20±0.80	2.18±0.54	40.08±9.97	42.95±10.69
SNP 10 µM	13.47±0.68	101.03±5.08	1.80±0.09	5.46±0.27	2.63±0.13	3.85±0.19	6.87±0.35	121.64±6.12	135.11±6.79
SNP 50 µM	4.10±0.11	21.54±0.59	0.40±0.01	1.28±0.04	0.48±0.01	0.64±0.02	0.85±0.02	25.18±0.69	29.28±0.80
SNP 100 µM	1.29±0.12	6.71±0.62	0.07±0.01	0.25±0.02	0.16±0.01	0.19±0.02	0.29±0.03	7.67±0.71	8.96±0.82

* 80 µmol/m^2/s for 14 days, ** 1200 µmol/m^2/s for 7 days, *** 1200 µmol/m^2/s for 14 days

Table S4. The content and production of CQAs in 30-days-old *Ib-rolB/C*-transgenic *I. batatas* callus cultures.

Callus line	CQAs content, mg/g DW									Dry weight (g/L)
	CGA	3,5-diCQA	3,4-diCQA	4,5-diCQA	3C-5CoQA	3F-5CQA	3C-5FQA	Total diCQAs	Total CQAs	
Control	0.56±0.05	3.23±0.08	0,18±0.01	0,12±0.01	0,06±0.00	0,18±0.02	0,22±0.03	3,99±0.56	4.55±0.47	14.67±0.29
IbB/C-1	0.93±0.05	6.29±0.09	0,25±0.01	0,21±0.01	0,12±0.01	0,22±0.01	0,53±0.01	7,62±1.10	8.55±0.92	12.44±0.05
IbB/C-2	0.69±0.06	5.37±0.12	0,08±0.01	0,13±0.01	0,11±0.01	0,15±0.01	0,34±0.04	6,19±0.95	6.89±0.79	9.00±0.01
	CQAs production, mg/L									
Callus line	CGA	3,5-diCQA	3,4-diCQA	4,5-diCQA	3C-5CoQA	3F-5CQA	3C-5FQA	Total diCQAs	Total CQAs	
Control	8.23±0.16	47.40±0.95	2.64±0.05	1.81±0.04	0.95±0.02	2.59±0.05	3.16±0.06	58.55±1.17	66.78±1.34	
IbB/C-1	11.62±3.23	78.17±21.74	3.16±0.88	2.59±0.72	1.47±0.41	2.76±0.77	6.55±1.82	94.70±26.34	106.32±29.57	
IbB/C-2	6.25±0.48	48.36±3.72	0.75±0.06	1.18±0.09	1.03±0.08	1.36±0.10	3.08±0.24	55.75±4.28	62.00±4.76	

Table S5. List of primer sequences used in this study.

Primer name (GenBank accession no.)	Forward (5′ to 3′)	Reverse (5′ to 3′)
Transgenic cell cultures screening		
*npt*II (AY818371)	TGATATTCGGCAAGCAGGCA	TTGTCACTGAAGCGGGAAGG
TEV 5'UTR (AY818371)	TCACCATTTACGAACGATAG C	-
I. batatas reference gene		
IbUBI (JX177358)	AGTCCACTCTCCACCTCGTC	CCTTTCCAGACTCATCCACC
qPCR analysis of biosynthetic genes		
IbPAL (MN823653, D78640)	ATGCTGACCGGAGAGAAGG	AACAGATAGGAAGAGGAGC C
IbC4H (GQ373157)	GAGTTCCGCCCAGAGAGG	CAAGATGTGGAGACTGAACT
Ib4CL (AB469557)	GTCCCTGTTGCGTTTGTGGT	TTATTGCGGCGTGGAAGGTG
IbHCT (AB576768)	GACGGAGGATCAAGAAAC CA	GGATCGCAGGTAATCGTTGT
IbHQT (AB035183, AB576769)	CACAGTCTACATTCTCCCCA	TCAGAAATCATACAAGAACT CC
qPCR analysis of cT-DNA genes		
Ib-rolB/C (KM052617)	CAATGCAATACCAGAGGAT T	GAAGCCATGAAACGGCGAC
ORF13 (KM052617)	CTTTATCGCTATCCAACTCA G	GACCGAACGCTCCAGATGC
ORF14 (KM052617)	CGTTGCCACCTTATCTCTTG	GCTGAAATGATCTCGTAGTT G
ORF17n (KM052617)	ATTGACGATATGCCAGCCG A	TGGAAAGCGTGATCGACAA C
ORF18/17n (KM052617)	AGAACTTAAAGGCACGGTC G	CTTGCATGGTAGGAGAGACG
